# Mapping and Spatial Pattern Analysis of COVID-19 in Central Iran Using the Local Indicators of Spatial Association (LISA)

**DOI:** 10.1186/s12889-021-12267-6

**Published:** 2021-12-08

**Authors:** Nahid Jesri, Abedin Saghafipour, Alireza Koohpaei, Babak Farzinnia, Moharram Karami Jooshin, Samaneh Abolkheirian, Mahsa Sarvi

**Affiliations:** 1 Remote Sensing & GIS Centre, Shahid Beheshti University, Tehran, Iran; 2 Department of Public Health, Faculty of Health, Qom University of Medical Sciences, Qom, Iran; 3 Occupational health & Safety Department, Faculty of Health, Qom University of Medical Sciences, Qom, Iran; 4 Department of Environmental Health Engineering, Faculty of Health, Qom University of Medical Sciences, Qom, Iran; 5 Department of Disease Control and Prevention, Qom Provincial Health Center, Qom University of Medical Sciences, Qom, Iran; 6 Department of Health Education and Promotion, School of Public Health, Tehran University of Medical Sciences, Tehran, Iran; 7 Student Research Committee, Qom University of Medical Sciences, Qom, Iran

**Keywords:** Coronavirus, COVID-19, Spatial analysis, Iran

## Abstract

**Background:**

Using geographical analysis to identify geographical factors related to the prevalence of COVID-19 infection can affect public health policies aiming at controlling the virus. This study aimed to determine the spatial analysis of COVID-19 in Qom Province, using the local indicators of spatial association (LISA).

**Methods:**

In a primary descriptive-analytical study, all individuals infected with COVID-19 in Qom Province from February 19^th^, 2020 to September 30^th^, 2020 were identified and included in the study. The spatial distribution in urban areas was determined using the Moran coefficient in geographic information systems (GIS); in addition, the spatial autocorrelation of the coronavirus in different urban districts of the province was calculated using the LISA method.

**Results:**

The prevalence of COVID-19 in Qom Province was estimated to be 356.75 per 100,000 populations. The pattern of spatial distribution of the prevalence of COVID-19 in Qom was clustered. District 3 (Imam Khomeini St.) and District 6 (Imamzadeh Ebrahim St.) were set in the High-High category of LISA: a high-value area surrounded by high-value areas as the two foci of COVID-19 in Qom Province. District 1 (Bajak) of urban districts was set in the Low-High category: a low-value area surrounded by high values. This district is located in a low-value area surrounded by high values.

**Conclusions:**

According to the results, district 3 (Imam Khomeini St.) and district 6 (Imamzadeh Ebrahim St.) areas are key areas for preventing and controlling interventional measures. In addition, considering the location of District 1 (Bajak) as an urban district in the Low-High category surrounded by high values, it seems that distance and spatial proximity play a major role in the spread of the disease.

## Background

In late 2019, a novel virus called severe acute respiratory syndrome coronavirus 2 (SARS-CoV-2) was identified in Wuhan, Hubei Province, China and, reported to be the cause of cases of pneumonia. The disease spread rapidly and after the epidemic in China, the cases increased throughout the world [1]. In February 2020, the World Health Organization (WHO) named the disease caused by the novel virus COVID-19 [2]. The United Nations has described coronavirus as a major social, humanitarian, and economic crisis that has many adverse effects on different countries [3]. Although people should take preventive measures to prevent the disease [4], it can still have indirect and potential impacts on public health in all groups of people [5]. Despite significant advances in disease control, communicable diseases are still of particular importance in epidemiology and community health [6].

One of the main applications of epidemiology is to facilitate the identification of geographical data affected by diseases and vulnerable groups that are at a higher risk of controlling diseases and being expose to risk factors. The identification of high-risk geographical areas and at-risk groups helps use appropriate health measures to reduce risk factors of infectious diseases [7]. The first step in analyzing the geographic data is to visualize it, especially in the form of geographic maps, which clearly show the pattern of geographical distribution of diseases, injuries, and deaths and pave the way for the creation of causal hypotheses [8]. In recent years, the use of geographic information systems (GIS) to determine geographical distribution patterns of diseases in medical and health sciences has increased significantly [9–12]. Determining the geographical distribution of diseases, the spatial study of care facilities and health services, determining the geographical boundaries of communities that are essential components of epidemiological and health studies are some of the applications of GIS in the field of health [13]. Spatial modeling in GIS is directly used to understand the differences in the spatial distribution of diseases and their relationship with environmental factors and health care system; as a result, GIS technology is currently a major tool in health research in the field of infectious diseases [14].

To determine spatial patterns of disease, the local indicators of spatial association (LISA) in the environmental GIS are very helpful. This model is a set of methods to describe and visualize spatial distributions, identify atypical locations or spatial outliers, determine patterns of spatial association, clusters or hot-spots, and propose spatial regimes or other shapes of spatial heterogeneity [15]. Due to the prevalence of the COVID-19 pandemic in the world including Iran and Qom Province, there is an urgent need to control and prevent the distribution of the disease. Qom Province was the first province in Iran in which COVID-19 was identified and reported. In late 2019, two cases of COVID-19 were identified and reported in this religious province home to several holy shrines [16]. People from around the world travel to the city of Qom to visit these special, religious sites. Therefore, it is necessary to determine the spatial pattern and geographical distribution of the disease in different districts and areas of Qom Province and identify its high-risk areas so that control and preventive measures can be carried out more purposefully. Therefore, this study aimed to determine the spatial distribution pattern analysis of COVID-19 in central Iran using the LISA method.

## Methods

**Fig. 1:**
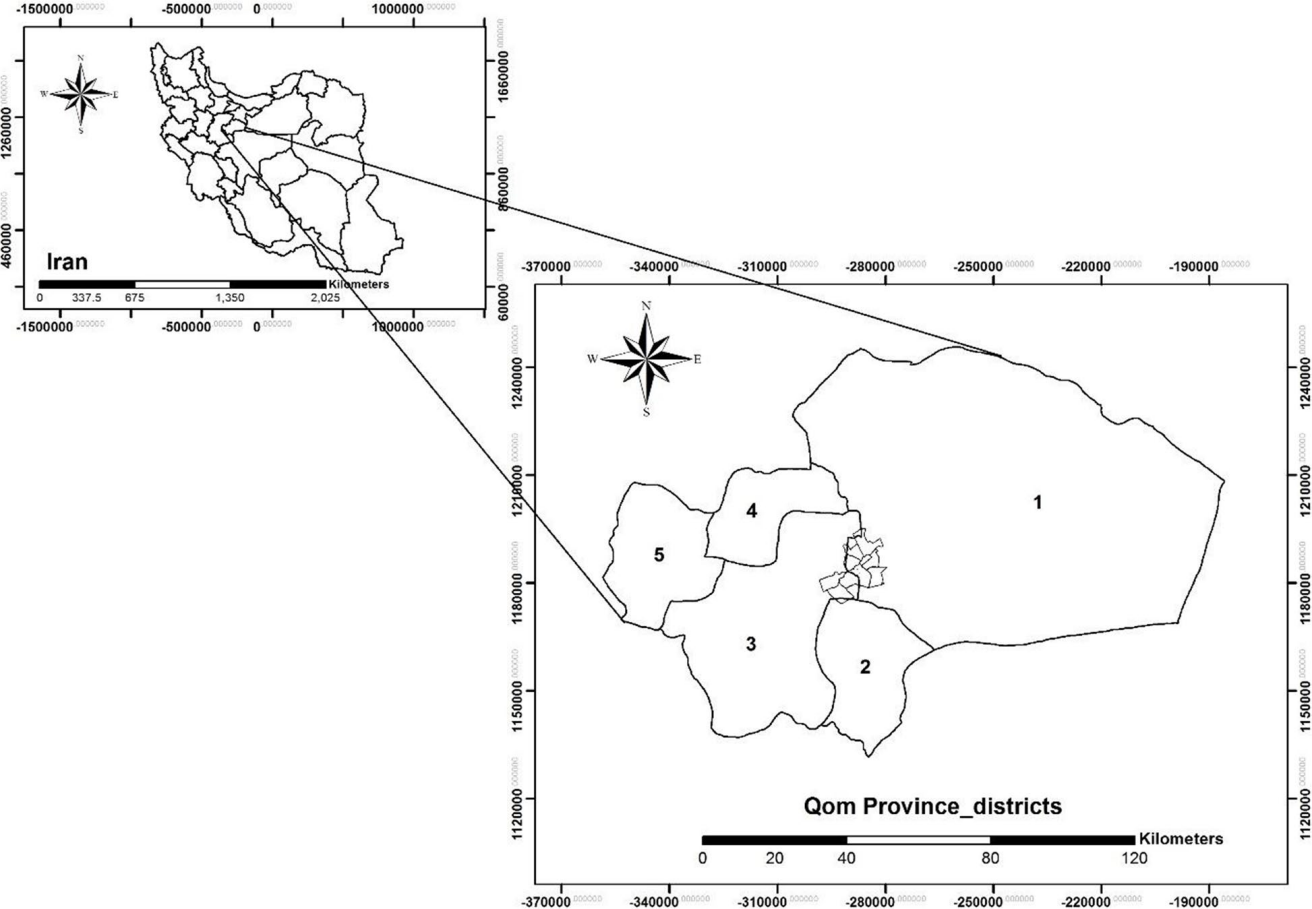
The study area; Qom Province in central Iran, rural districts (1: Markazi, 2: Kahak, 3: Salafchegan, 4: Jafarabad and 5: Khalajestan) and urban district (The city of Qom)

Qom Province is adjacent to some of the most important provinces in Iran (such as Tehran, Isfahan, Markazi and Semnan) [17]. Geographically, this province includes five rural districts: Markazi, Salafchegan, Kahak, Khalajistan and Jafarabad (Fig. 1). The city of Qom, as a subset of the Markazi district, is the only urban area of ​​the province with eight urban districts. These districts include: District 1 (Bajak), District 2 (Niroogah), District 3 (Imam Khomeini St.), District 4 (Janbilabad-Salarieh), District 5 (Jamkaran), District 6 (Imamzadeh Ebrahim St.), District 7 (Holy shrine), and District 8 (Pardisan).

In a primary descriptive-analytical study, all individuals infected with COVID-19 (n=4388) in Qom Province, from February 19^th^, 2020 to September 30^th^, 2020 were identified and included in the study. The data of patients were recorded through the emergency medical records of hospitals under the supervision of Qom University of Medical Sciences. The population of the area was prepared separately according to the postal address of patients in Qom Province and the analysis was finally performed in ArcGIS 10.6 mapping and modeling software.

After collecting the data in Excel 2010 (Microsoft, https://www.microsoft.com) and entering the data in ESRI ArcGIS 10.6 software (http://www.esri.com/arcgis), point density was used to show the spatial distribution of COVID-19 patients in all rural and urban districts of the province.

First, the incidence of COVID-19 infection during the study period was calculated. Then, the incidence rate (IR) was mapped on urban and rural digital maps based on a scale of 1/100,000. All analyses were performed by rural and urban-level districts, as basic units of analysis. In addition, the spatial distribution of the disease in urban areas of the province was investigated using Moran's coefficient. According to the Moran coefficient, there are three fundamental spatial point patterns: complete spatial randomness (CSR), regularity, and clustering [18]. Furthermore, the spatial autocorrelation of the novel coronavirus in the different urban districts of the province was calculated using the local indicators of spatial association (LISA) analysis. This index explains the spatial relationship pattern of a spatial parameter in the neighborhood. With the help of this statistic, points with low or high-values ​​that are distributed in clusters or values ​​with high value differences (with a random pattern) can be displayed [19].

To identify the spatial clustering of each feature, local spatial autocorrelation is mostly used. The local spatial autocorrelation is determined from the following formula:

1 $${I}_i=\frac{X_i-\overline{X}}{{\mathrm{S}}_i^2}\sum_{j=1,j\ne i}^n{W}_{i,j}\left({X}_i-\overline{X}\right)$$

*xi*: characters of parameter i
$$\overline{X}$$
the mean of parameters
*wi*, the matrix of weights that in some cases is equivalent to a binary matrix with ones in position i,j

The value of ${\mathrm{S}}_i^2$ is obtained from the following formula:

2 $${S}_i^2=\frac{\sum_{j=1,j\ne i}^n{\left({X}_i-\overline{X}\right)}^2}{\mathrm{n}-1}$$

n: total number of features

The standard *Z*_*ij*_ score is obtained from the following equation:

3 $${Z}_{ij}=\frac{I_i-\mathrm{E}\left[{I}_i\right]}{\sqrt{V\left[{I}_i\right]}}$$

The value of E[*I*_*i*_] and *V*[*I*_*i*_] is obtained from the following formula:

4 $$\mathrm{E}\left[{I}_i\right]=-\frac{\sum_{j=1,j\ne i}^n{W}_{ij}}{\mathrm{n}-1}$$

5 $$V\left[{I}_i\right]=E\left[{I}_i^2\right]-E{\left[{I}_i\right]}^2$$

It is possible to display points in this statistic with four modes: High-High (HH), High-Low (HL), Low-High (LH), Low-Low (LL). The interpretation of these four categories is shown in Table 1. If *I*_*i*_ is positive and significant, it indicates that the area is surrounded by similar-value areas: High-High (HH) and Low-Low (LL). If *I*_*i*_ is negative and significant, it means the area is surrounded by different-value areas: High-Low (HL) and Low - High (LH).

**Table 1:** The interpretation the results of the local indicators of spatial association (LISA).

Categories	Autocorrelation	Interpretation
HH	Positive	Cluster - A high value area surrounded by high value areas.
HL	Negative	Complete spatial randomness - A high value area surrounded by low value areas
LL	Positive	Cluster - A low value area surrounded by low value areas.
LH	Negative	Complete spatial randomness - a low value area surrounded by high values

## Results

### The epidemiological features of patients

A total of 4,388 patients with COVID-19 were diagnosed from February 19^th^, 2020 to September 30^th^, 2020 in Qom Province located in central Iran, most of whom were natives and residents of urban areas of the province. Also, most cases were men over 50 years of age (Table 2).

**Table 2:** Some epidemiological characteristics of patients with COVID-19 in Qom Province, Central Iran from February 19, 2020 to September, 2020

Cases		N	(%)
Gender	Male	2340	53.32
	Female	2048	46.68
Age groups (Year)	10>	90	2.04
	30-10	348	7.94
	50-30	1311	29.87
	≥50	2639	60.15
Endemisity situation	Indigenous	4340	98.9
	None-indigenous	48	1.1
Residency status	Urban	4210	97.00
	Rural	130	3.00
	Total of indigenous	4340	100

The prevalence of COVID-19 infection in Qom Province was estimated to be 356.75 per 100,000 populations in the studied period.

**Fig. 2:**
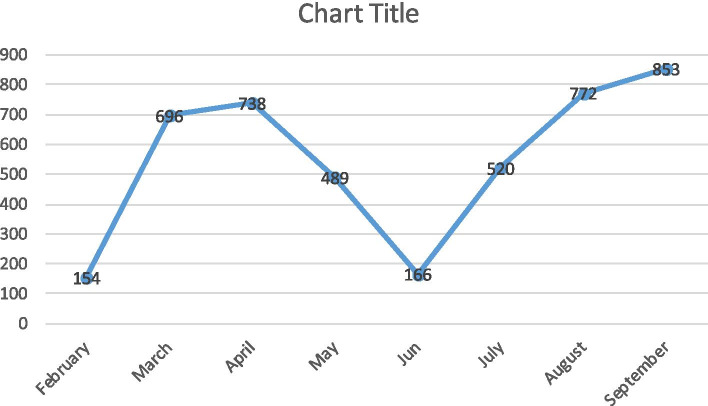
The monthly incidence of COVID-19 disease in Qom Province, Central Iran from February 19^th^, 2020 to September 30^th^, 2020

The monthly incidence of COVID-19 disease in Qom Province shows that the frequency of cases increased from February to April, after the reduction of the disease in two months (Fig. 2).

### The spatial distribution of COVID-19 infection

**Fig. 3:**
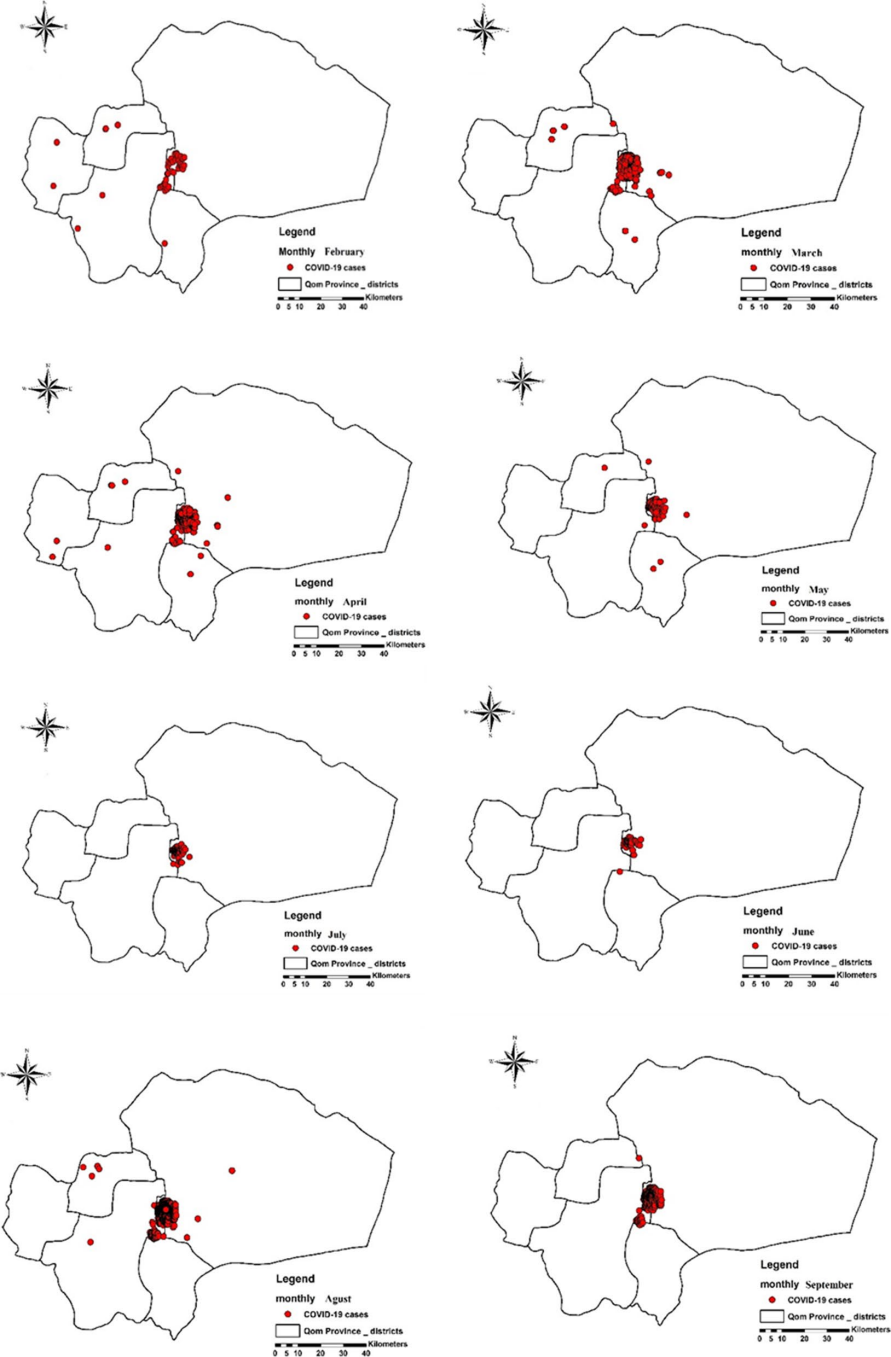
Monthly Spatial distribution maps of COVID-19 disease in Qom Province, Central Iran from February 19^th^, 2020 to September 30^th^, 2020

**Fig. 4:**
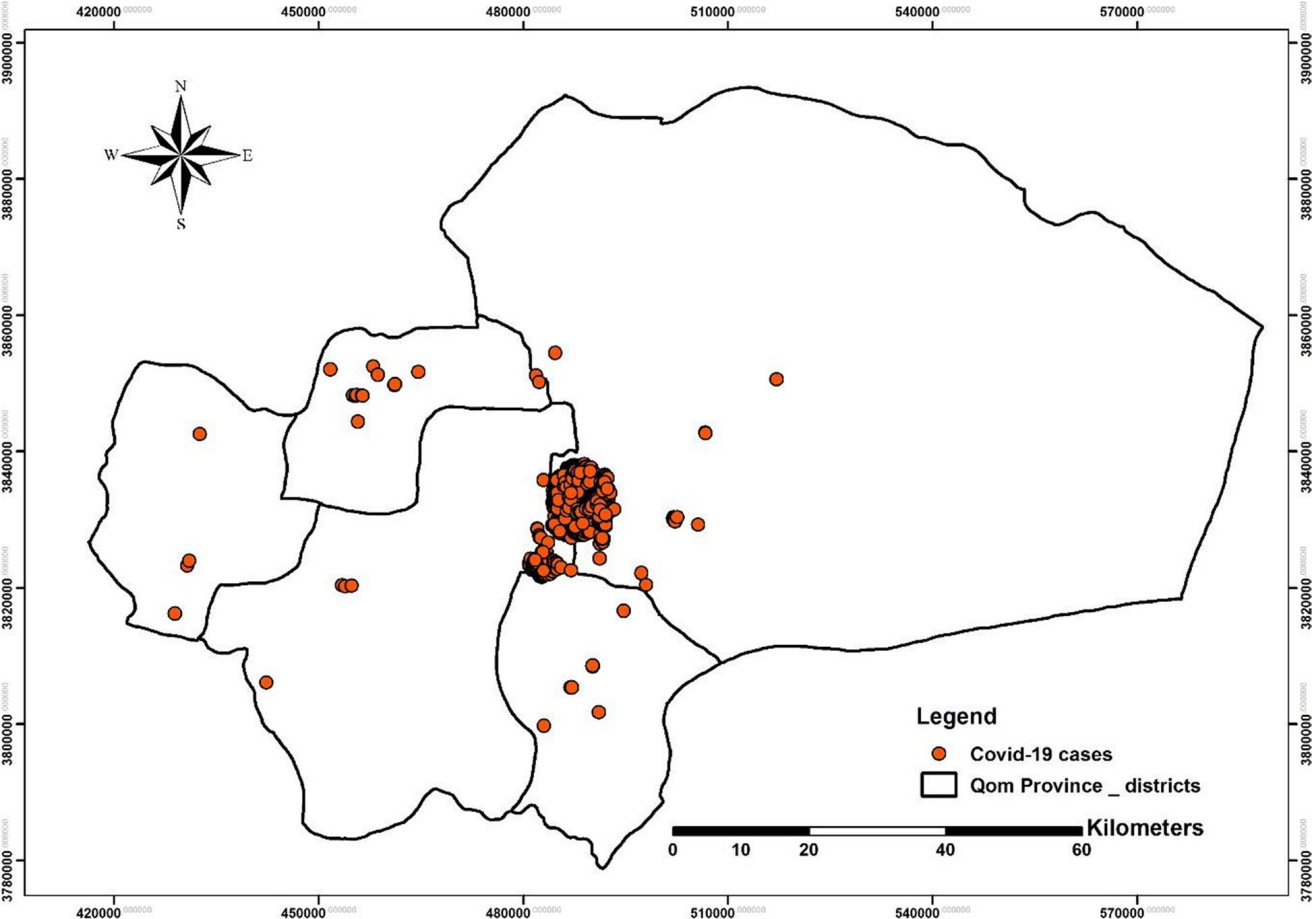
Spatial distribution maps of COVID-19 disease in rural and urban areas of Qom Province, Central Iran from February 19^th^, 2020 to September 30^th^, 2020

The spatial distribution maps of COVID-19 disease in Qom Province show that most cases (97%) of the disease occurred in the Markazi district and the city of Qom with few cases (3%) reported from other rural districts (Fig. 3,4). The prevalence of COVID-19 disease in urban districts was 350.49 per 100,000 populations and 4.51 per 100,000 populations in rural areas. The prevalence of the disease in the urban districts shows that District 7 (Holy shrine), District 4 (Janbilabad-Salarieh), and District 6 (Imamzadeh Ebrahim St.) had the highest incidence of the disease in the studied period in Qom (Table 3).

**Table 3:** Prevalence of COVID-19 infection in urban areas of Qom Province, Central Iran from February 19, 2020 to September, 2020

Urban districts	Population	Cases	Incidence/100,000
1	78401	243	309.94
2	192060	671	349.37
3	189708	642	338.41
4	171363	808	471.51
5	192755	642	333.06
6	213356	930	435.89
7	41625	227	545.34
8	121890	225	184.59

### The point pattern analysis of COVID-19 in Qom Province

**Fig. 5:**
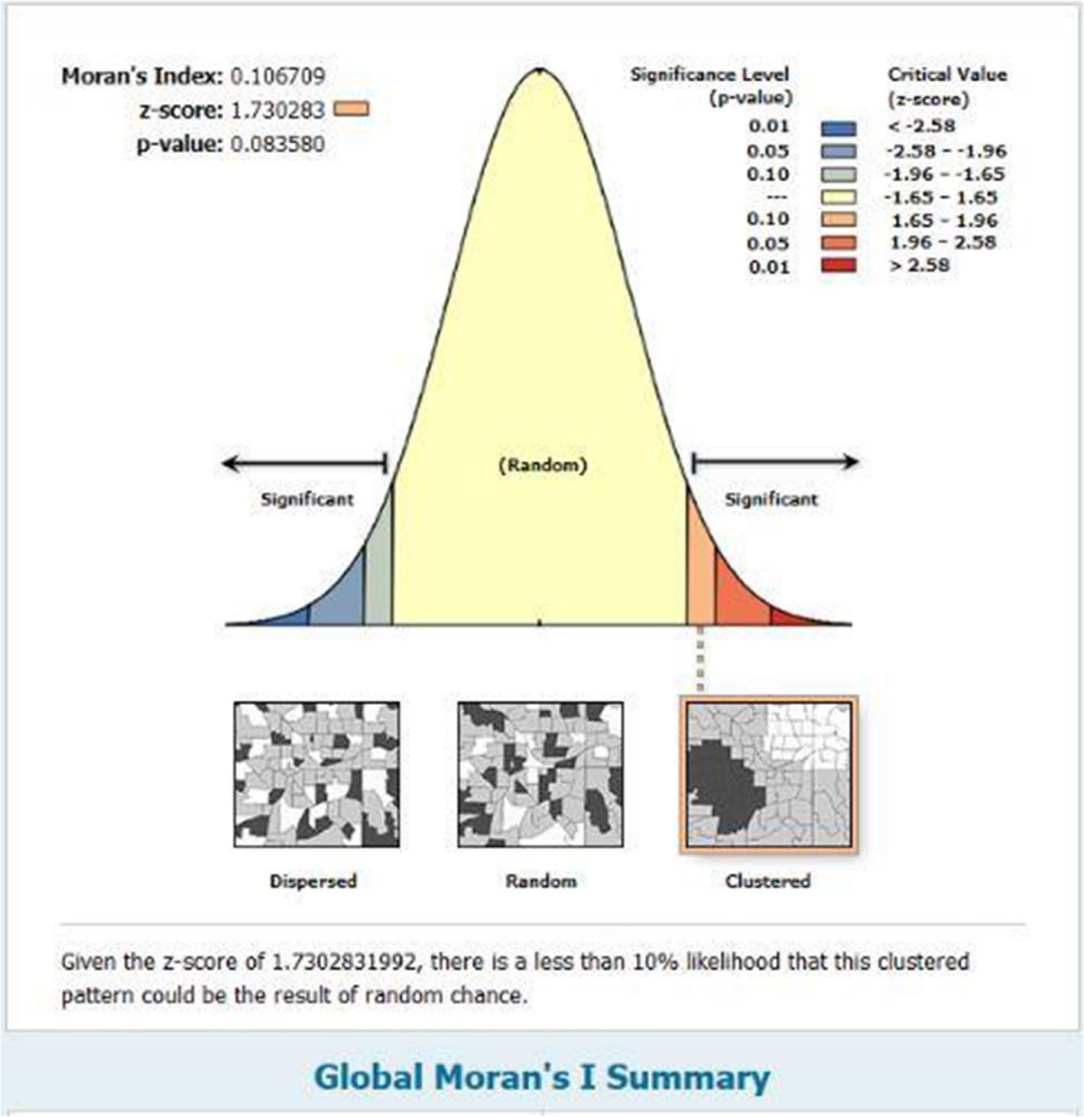
The point pattern analysis of COVID-19 in Qom province, from February 19^th^, 2020 to September 30^th^, 2020

With the spread of the novel coronavirus in Qom Province since February 19^th^, 2020, to determinate the point pattern analysis of COVID-19, Moran's Index was calculated (Moran's Index: 0.106709, Z-score: 1.730283 and p-value: 0.083580 with 90% confidence interval). The pattern of spatial distribution of the prevalence of COVID-19 disease in Qom was then clustered (Fig. 5), indicating the high prevalence of the disease in some areas of Qom, including urban districts 7, 4, and 6 respectively (Table 3).

### The spatial autocorrelation of the novel coronavirus using the local indicators of spatial association (LISA)

**Fig. 6:**
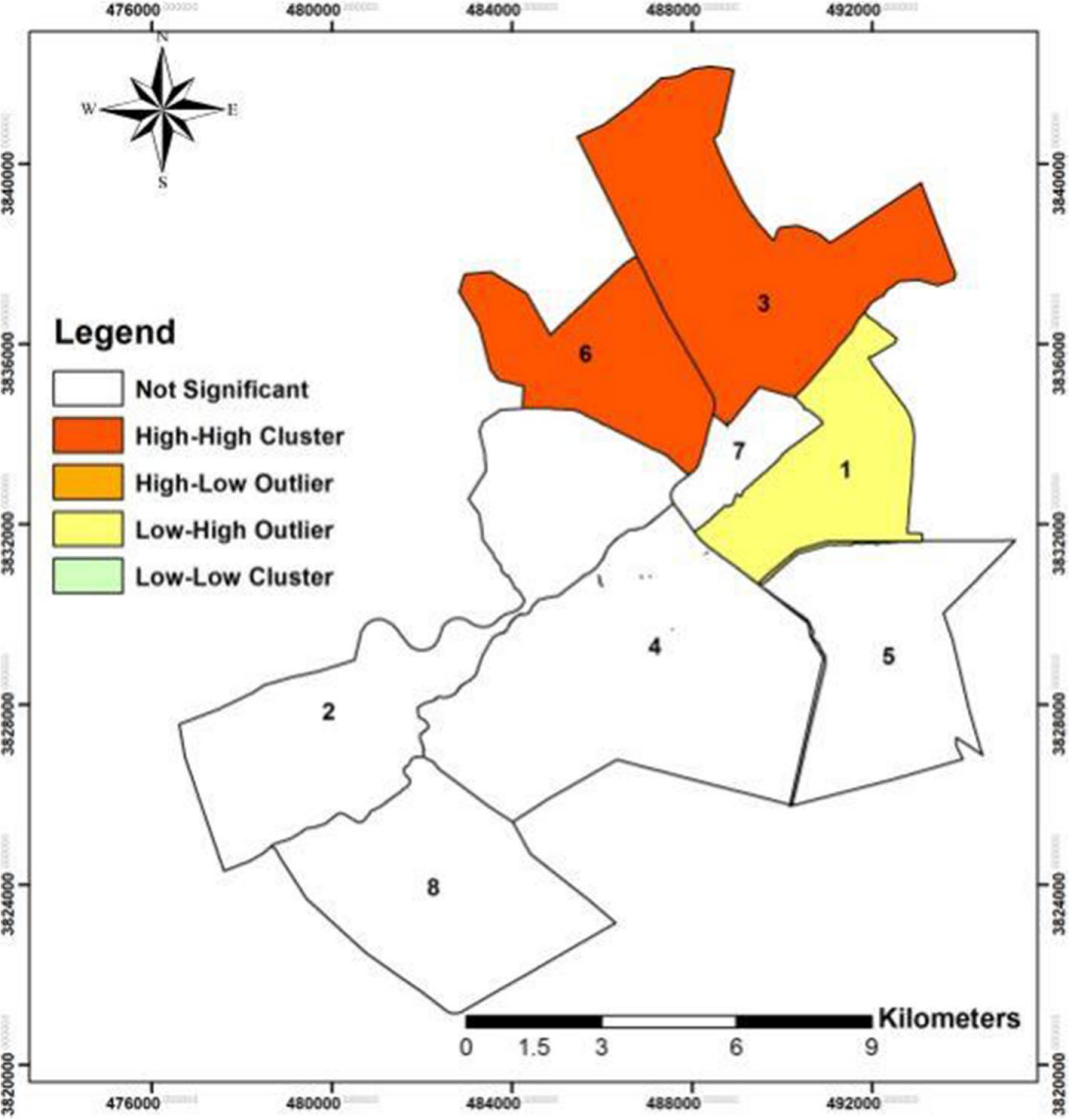
The spatial autocorrelation of COVID-19 disease using the local indicators of spatial association (LISA) in urban districts of the city of Qom, from February 19^th^, 2020 to September 30^th^, 2020

Based on the result of the analysis, the incidence of COVID-19 infection cases in District 3 (Imam Khomeini St.) and District 6 (Imamzadeh Ebrahim St.), as two urban districts of of Qom Province was set in the HH category: a high-value area surrounded by high-value areas (areas with a high incidence of patients with COVID-19). Thus, according to the results of LISA analysis, these districts were the two foci of COVID-19 in Qom Province (Fig. 6). Also, autocorrelation in these two urban districts is positive and includes more than one-third of the population of Qom (33.55%).

In addition, based on the results of LISA analysis, District 1 (Bajak) of urban districts was set in the LH category: a low-value area surrounded by high-values. This district is located in a low-value area surrounded by high-value areas (Fig. 6).

## Discussion

The coronavirus pandemic is currently disrupting the daily life of people around the world, and it is thus necessary to address this health issue and study it in terms of geographical features [20]. Based on the findings of present the study, the prevalence of COVID-19 infection in Qom Province was estimated to be 356.75 per 100,000 population in a period of time from February 19^th^, 2020 to September 30^th^, 2020. Following the ongoing spread of COVID-19 in the world, the outbreak of the disease was confirmed in Iran and the city of Qom on February 20^th^, 2020 [16]. The monthly incidence of COVID-19 in Qom Province in the studied period shows that the frequency of cases increased substantially from February to April; after the reduction of cases for two months, the number of cases increased again in the following months. It seems that the observance of health protocols including social distancing, regular hand washing, use of masks and gloves, home quarantine by people, and closure of pilgrimage and tourism centers could reduce the incidence of the disease in May and June [21]. In China, the mortality rates have decreased at certain times of the year in accordance with protocols. For instance, due to the observance of health in early March, there was a significant reduction in the number of cases and deaths [22]. The results of this study indicate that the pattern of the spatial distribution of the prevalence of COVID-19 disease in Qom was clustered. Furthermore, the incidence of COVID-19 cases in District 3 (Imam Khomeini St.) and District 6 (Imamzadeh Ebrahim St.), as two urban districts of Qom Province was set in the HH category of LISA as two foci of COVID-19 in the province. This finding demostrates that the incidence of the disease in these two districts was higher than the average (values ​​above average), and these two districts were also surrounded by high-value areas (areas with a high incidence of patients with COVID-19). Moreover, autocorrelation in these two urban districts was positive including more than one-third of the population of Qom. Also, based on the results of LISA analysis, District 1 (Bajak) of urban districts was set in the LH category. Because of its spatial interaction with areas at a high risk of coronavirus transmission, precautionary and preventive measures aiming to control coronavirus infection in this area needs intensified. It seems that one of the most important factors in the spatial spread of coronavirus in Qom Province was the centralization of population distribution in the city of Qom and around the holy shrine, which in the coronavirus pandemic situation caused the spread of this communicable disease.

In a study conducted in Hubei Province, China, the results of the spatial pattern of the Moran coefficient showed spatial clusters with an increasing trend and a sudden change, revealing that the high concentration of population caused the outbreak of the Corona-virus in the province [23]. In addition, the findings of spatial pattern analysis of COVID-19 in Iran showed that dominant adjacent provinces with old age structures and higher mean temperatures were the most susceptible provinces to have higher cases of COVID-19. Likewise, areas in the north of Iran, particularly Qom, Marzaki, Mazandaran, and Semnan provinces have experienced higher prevalence of the disease [24]. In comparison with Iran, research shows that the spatial and temporal pattern of COVID-19 confirmed cases in China has followed four patterns: Hubei was the initial core region, the eastern provinces adjacent to Hubei formed the second concentrated pattern, the western provinces adjacent to Hubei and the northeastern and southeastern provinces separated from Hubei by one province belonged to the third distribution pattern, and the remaining provinces in the north, south, and west showing sporadic distribution patterns formed the fourth pattern [22].

## Conclusion

According to the results, District 3 (Imam Khomeini St.) and District 6 (Imamzadeh Ebrahim St.) can be regarded as key areas for preventing and controlling the disease as well as implementing interventional measures. In addition, considering the placement of District 1 (Bajak) of urban districts in the LH category surrounded by high-value areas, it seems that distance and spatial proximity plays a major role in the spread of the disease. Therefore, it is recommended to adopt preventive matures such as social distancing in high-traffic, heavily populated areas, especially District 3 (Imam Khomeini St.) and District 6 (Imamzadeh Ebrahim St.) as the two foci of COVID-19 in the city of Qom, more than before to cut the chain of disease transmission.

## Data Availability

The datasets used and/or analysed during the current study are available from the corresponding author on reasonable request.

## Abbreviations

LISA: Local indicators of spatial association
SARS-CoV-2: Severe acute respiratory syndrome coronavirus 2
WHO: World Health Organization
